# Abrasive Wear of High-Carbon Low-Alloyed Austenite Steel: Microhardness, Microstructure and X-ray Characteristics of Worn Surface

**DOI:** 10.3390/ma14206159

**Published:** 2021-10-17

**Authors:** Michail Nikolaevich Brykov, Taisiia Oleksandrivna Akrytova, Michail Jurievich Osipov, Ivan Petryshynets, Viktor Puchy, Vasily Georgievich Efremenko, Kazumichi Shimizu, Maik Kunert, Olaf Hesse

**Affiliations:** 1 Welding Department, Zaporizhzhia Polytechnic National University, Zhukovsky 64, 69063 Zaporizhzhia, Ukraine; akritova7@ukr.net (T.O.A.); mosipov61@ukr.net (M.J.O.); 2 Division of Metallic Systems, Institute of Materials Research, Slovak Academy of Sciences, Watsonova 47, 04001 Kosice, Slovakia; ipetryshynets@saske.sk (I.P.); vpuchy@saske.sk (V.P.); 3 Physics Department, Pryazovskyi State Technical University, Universitetska 7, 87555 Mariupol, Ukraine; vgefremenko@gmail.com; 4 Mechanical Engineering Research Unit, College of Design and Manufacturing Technology, Muroran Institute of Technology, Mizumoto-cho 27-1, Muroran 050-8585, Hokkaido, Japan; shimizu@mmm.muroran-it.ac.jp; 5 SciTec Department, Ernst-Abbe-Hochschule Jena, Carl-Zeiss-Promenade 2, 07745 Jena, Germany; maik.kunert@eah-jena.de (M.K.); olaf.hesse@eah-jena.de (O.H.)

**Keywords:** high-carbon steel, abrasive wear, retained austenite, mechanically-induced martensite transformation, work-hardening, worn surface, microhardness, XRD, microstructure, SEM

## Abstract

A high-carbon, high-silicon steel (1.21 wt% C, 2.56 wt% Mn, 1.59 wt% Si) was subjected to quenching from 900 and 1000 °C, resulting in microstructures containing 60 and 94% of retained austenite, respectively. Subsequent abrasive wear tests of quenched samples were performed using two-body abrasion and three-body abrasion testing machines. Investigations on worn surface and subsurface were carried out using SEM, XRD, and microhardness measurement. It was found that the highest microhardness of worn surface (about 1400 HV0.05) was achieved on samples quenched from 900 °C after three-body abrasion. Microhardness of samples after two-body abrasion was noticeably smaller. with a maximum of about 1200 HV0.05. This difference correlates with microstructure investigations along with XRD results. Three-body abrasion has produced a significantly deeper deformed layer; corresponding diffractograms show bigger values of the full width at half maximum parameter (FWHM) for both α and γ alone standing peaks. The obtained results are discussed in the light of possible differences in abrasive wear conditions and differing stability of retained austenite after quenching from different temperatures. It is shown that a structure of metastable austenite may be used as a detector for wear conditions, as the sensitivity of such austenite to phase transformation strongly depends on wear conditions, and even small changes in the latter lead to significant differences in the properties of the worn surface.

## 1. Introduction

Friction and wear are responsible for about 20% of total world energy consumption [1]. The most aggressive type of wear is abrasive wear (AW); it is responsible for significant losses of material in such industries as the mining sector [2,3].

AW is a process of multiple chaotic interactions of harder asperities with softer surface under applied load. Even today, a prediction of the summative superposition of the results of all individual interactions is not possible. However, the total amount of wear-related material loss is approximately constant for a sufficiently long sliding distance and stable wear conditions. This is known as the Reye [4]–Archard [5,6]–Khrushchov [7] wear law and is fundamental knowledge used to determine the wear rate or wear resistance of materials, and in particular, the wear conditions.

There is one major issue concerning AW tests or wear modes in different wear applications: the loss of material is very sensitive for wear conditions [8]. Even minor changes in the latter lead to a significant alteration in the material loss as a result of wear. Load, nature, shape and/or size of abrasive grits, humidity, temperature, and so on appear as such wear conditions. Therefore, the very first task when investigating wear resistance is to provide stable wear conditions during testing.

There are a number of examples in laboratory practice when efforts are made to standardize AW conditions, as shown in [9]. This allows obtaining comparable and reproducible data about the AW rate or AW resistance of materials. When it comes to practical wear applications, “standardization” of wear conditions is hardly possible, and it is important to somehow characterize particular wear conditions. Attempts have been made to predict wear by modelling [6,10,11]. Additionally, some special material is needed which would be sensitive enough to reflect minor deviations in the wear environment.

Actually, any metal or alloy may be used as such sensitive material. A hydrostatic stress state is formed in front of and below the moving particles in the course of wear [12]. As a result, the material in the subsurface layer undergoes severe plastic deformation of several hundred percent [12,13,14]. This plastic deformation leads to work hardening, local breakage of interatomic bonds, and detachment of wear particles. The average level of hardening at the very surface or in the sub-surface layer may be used as an indicator of wear “severity” or wear mode. However, this approach has some drawbacks. The breakage of interatomic bonds occurs only after maximum possible work hardening is achieved in a given micro-volume of a material. Therefore, in any wear mode, the degree of hardening in the vicinity of a micro-crack would be the same. This is because the maximum possible degree of work hardening (i.e., maximum possible dislocation density) is the same for iron alloys [15]. Hence it is hardly possible to distinguish the difference in wear modes solely using degree of hardening of worn (sub-)surface as an indicator. From the other side, a combination of the degree of hardening with some other criterion may be useful. This additional criterion may be a structure state of a material before and after AW. Ideally, a material should be able to change its structure during wear depending on the wear mode.

The transformation of metastable austenite to martensite under mechanical load can be used as a phenomenon to distinguish between wear conditions. This effect is being extensively used to increase the wear resistance of steels and cast irons subjected to cavitation [16] and AW [17,18,19,20]. Transformation is possible when austenite undergoes deformation (bulk [21,22] or in a thin surface layer [17,18]) in the temperature range between the martensite start temperature (M_s_) and temperature M_d_. At temperatures above M_d_, austenite cannot be transformed at any degree of deformation [23,24]. In order to provide the maximum possible extent of phase transformation, it is necessary to deform austenite at a temperature that is close to M_s_. According to these considerations, it is necessary to obtain austenite with an M_s_ point near 20 °C in order to provide the highest degree of transformation during AW at room temperature (~20 °C). This will ensure an increased amount of retained austenite which could be transformed into martensite under wear.

Novel low-alloy high-carbon steel X120Mn3Si2 was proposed recently as a material with higher wear resistance against AW [25,26]. This steel remains almost fully austenitic after quenching from 1000 °C. The degree of phase transformations in a thin subsurface layer in the course of AW is high enough due to the very high instability of retained austenite. The hypothesis that is worked out in the present paper is that differences in AW conditions will have a noticeable influence on the degree of structural changes and properties of the worn surface of this steel. Therefore, the task is to investigate the AW behavior and to characterize the worn surface of X120Mn3Si2 steel under different AW testing conditions.

## 2. Materials and Methods

The studying material was X120Mn3Si2 steel of chemical composition: 1.21 wt% C, 2.56 wt% Mn, 1.59 wt% Si. The steel was melted in vacuum furnace (COMTES FHT, Dobřany, Czech Republic), cast into blocks, forged, and rolled to strips of 5 mm thickness. After annealing at 900 °C and slow cooling, the strip was cut into specimens which were used for further heat treatment, wear tests, microhardness measurement, microstructure, and XRD characterization.

Samples for AW tests were exposed to two heat treatment regimes: quenching from 900 °C and quenching from 1000 °C. These treatments were chosen to provide predominantly austenitic structure of samples with slightly different stabilities of retained austenite. After quenching from 900 °C, austenite is less stable, and there are some martensite plates and undissolved carbides present in the structure. After quenching from 1000 °C, the structure is almost fully austenitic, and austenite is a bit more stable because the carbides are fully dissolved and the carbon content of the austenite is larger. M_s_ of these samples are estimated to be 30–50 °C for quenching from 900 °C and 10–30 °C for quenching from 1000 °C [26]. Actually, the precise values of M_s_ are not important themselves, but their slight shift relative to each other is. It is expected that this shift, as a result of changed austenite chemical composition, should have some influence on wear rate and properties of surface zones of samples after AW in different regimes. This presumption is grounded on two known facts: (1) the stability of retained austenite to mechanically induced martensite transformation depends on carbon content [23]; (2) the wear resistance of retained austenite depends on its stability [27,28].

Values of microhardness for X120Mn3Si2 after quenching from 900 °C and 1000 °C were 420 HV and 220 HV, respectively.

In addition to samples for AW tests, two samples were quenched from 800 °C and 1000 °C to provide reference values for martensite and austenite XRD peaks.

After heat treatment, the specimens were ground to remove the decarburized layer.

AW tests were performed using two different testing installations. Two-body AW tests were made using NUS-ISO3 testing machine (SUGA TEST INSTRUMENTS, Tokyo, Japan). A flat specimen of 4 × 50 × 50 mm^3^ size is pressed by force of 19 N to a wheel with attached abrasive paper (180 mesh SiC). The specimen moves 30 mm back and 30 mm forth, making one cycle. After every cycle, the wheel rotates by 0.9 degrees to prevent clogging of the friction interface and provide fresh abrasive. After each full wheel rotation (400 cycles), the abrasive paper is changed. The total number of cycles per one test is 1600, which corresponds to 96 m of wear path. The weight loss of a sample after each test was measured by electronic balance RADWAG AS 60/220.R1 (RADWAG, Radom, Poland) with accuracy of 0.1 mg. Each result represents the average of three identical tests. This wear mode is further designated as A mode.

For three-body AW tests, the testing installation built in Zaporizhzhia Polytechnic National University (Zaporizhzhia, Ukraine) was used as described in [29]. The abrasive was silicon carbide with grit size of about 0.60–1.00 mm. The weight loss was measured using a balance with an accuracy of 0.1 mg. Each value of weight loss represents the average of three measurements. This wear mode is further designated as B mode.

According to heat treatment and testing procedures, there are four combinations of sample designations: A900—quenching from 900 °C + A wear mode; A1000—quenching from 1000 °C + A wear mode; B900—quenching from 900 °C + B wear mode; B1000—quenching from 1000 °C + B wear mode.

Cross-sections of samples were prepared according to standard metallographic procedure by polishing the sample’s surface with SiC sandpaper and alumina aqueous solutions, with further etching by 3%-nital reagent. Cross-sections of worn samples and worn surfaces were investigated using SEMs JSM-7000F (JEOL, Tokyo, Japan), Vega3 (TESCAN, Brno, Czech Republic), and Ultra 55 (CARL ZEISS, Jena, Germany). Microhardness of worn surfaces was determined by testers FM-300 (FUTURE TECH CORP., Kawasaki, Japan) and computer controlled Wilson^®^ Hardness tester (BUEHLER, Esslingen am Neckar, Germany) at 0.05 kg load. Phase composition of as-quenched samples and sample surfaces after AW was determined by XRD analysis using D8 Discover (BRUKER, Billerica, MA, USA) diffractometer with CuKα-radiation. XRD measurements of heat-treated specimens were performed on carefully ground and polished surfaces to minimize mechanical deformation of surface layers. The volume fractions of austenite and martensite in as-quenched samples were determined using Rietveld analysis [30]. The volume fractions of austenite VF_RA_ of the worn specimen were obtained using traditional single peak methods [31,32]:

(1) $${\mathrm{VF}}_{\mathrm{RA}}=\frac{100\%}{1+G\left(\frac{I_{\alpha }}{I_{Y}}\right)}$$

where h, k, l—indices of crystallographic planes, λ—wavelength of X-radiation, Iα, Iγ—integrated intensities of diffraction peaks (110)α-Fe and (111)γ-Fe, respectively; and G—coefficient corresponding to different combinations of peaks, namely: 2.46 for Iα(200)/Iγ(200); 1.32 for Iα(200)/Iγ(220); 1.78 for Iα(200)/Iγ(311); 1.21 for Iα(211)/Iγ(200); 0.65 for Iα(211)/Iγ(220); 0.87 for Iα(211)/Iγ(311) [31].

**Figure 1:**
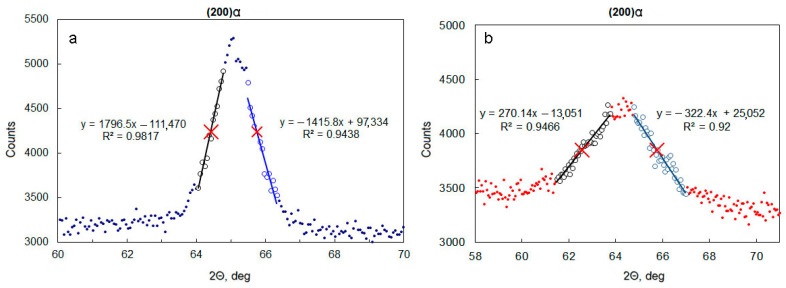
Examples of determining Full Width on Half Maximum (FWHM) for (200)α peak: (**a**) quenching from 800 °C; (**b**) B1000.

The XRD of worn specimens was performed on the as-worn surfaces. Plastic deformation of material as a result of AW causes peak broadening in XRD patterns. There are two ways to estimate peak width: full width on half maximum (FWHM) and integral breadth (IB). Firstly, the XRD pattern should be refined, but in case of worn specimens, the refinement of the whole pattern is hard to implement [33]. It is possible to estimate the FWHM of a given peak in the XRD pattern of worn material without fitting the whole diffractogram. Two slopes of a peak may be approximated by linear functions using limited sets of dots on an XRD pattern (Figure 1). Half of maximum would be the distance between the maximum value of intensity for a given peak and the mean intensity of a background. Intersections of linear approximations with half of the maximum level are two dots (red crosses on Figure 1), which were used to determine FWHM.

## 3. Results and Discussion

### 3.1. Abrasive Wear Tests

Results of abrasive wear test are shown in Table 1. It is seen that absolute values of wear rates for three-body wear mode (B900 and B1000 samples) are two orders of magnitude greater than those for two-body wear mode (A900 and A1000 samples). This may be explained by taking into account the difference in pressure and abrasive grit size applied in each mode. The pressure in mode B is about three times higher than that in mode A, (i.e., 5 MPa and approximately 1.6 MPa, respectively). In addition, the grit size in mode B is 0.6–1.0 mm, against 0.09 mm in mode A. 

Another significant point that attracts attention is the reverse behavior of wear rates for 1000 and 900 samples for A and B modes. This fact can be explained as follows.

After quenching from 1000 °C, the M_s_ temperature is slightly lower than that of quenching from 900 °C. Therefore, the 1000 °C-quenched austenite is more stable to mechanically induced transformation than the 900 °C-quenched one. Apparently, the difference in austenite stability is the main reason for the higher wear rate of the 1000 °C sample in A mode. The pressure in A mode is relatively low, and it is not enough to transform 1000 °C-quenched austenite to martensite at the maximum extent. The higher the austenite stability, the lower is the transformation degree, and the higher is the mass loss.

Concerning wear in B mode, the mass loss for the 1000 °C sample is actually less than that of 900 °C sample. A possible reason is the presence of cementite in 900 °C samples, which tends to brittle fracture during abrasive wear at high pressure [34].

In order to understand the reasons for different wear behaviors of instable austenite in different abrasive wear conditions, microstructure investigation of near-surface regions, microhardness measurements, and X-ray diffraction analysis of worn surfaces have been performed.

### 3.2. XRD of Worn Surface

X-ray diffraction study showed that XRD patterns of all samples consisted of the peaks belonging to α-Fe and γ-Fe (austenite). No peaks of carbides were found. Table 2 summarizes the FWHM values calculated for separately standing α and γ peaks for all tested samples. In addition, the XRD data for X120Mn3Si2 steel quenched from 800 °C and 1000 °C are presented. These data serve as the base for comparison of α and γ peaks in the initial condition (as-quenched) and after abrasive wear.

It is seen that the FWHM for B mode samples is always bigger than for A mode samples. This may be explained by the higher wear stress in B mode. Another important point is that the most widened peaks are (200)α and (200)γ. Line broadening for these peaks in comparison with the as-quenched state is maximal among other peaks in the corresponding group (α or γ). 

FWHM values of γ-peaks for A900 and B900 samples were not estimated because the heights of the peaks were so low that they were merely bigger than background level. 

Table 3 shows the summarized results of XRD investigation of worn surfaces for all samples. 

According to data given in Table 2 and Table 3, some general observations arise:

- Values of FWHM for all peaks (see Table 2) are greater for B mode samples than for A mode samples if comparison is made for a certain quenching temperature (900 °C or 1000 °C);
- Relative amounts of transformed austenite (see Table 3) are also greater for B-mode samples than that for A-mode samples at any given quenching temperature;
- Among all γ peaks, for both B1000 and A1000 samples (see Table 2), the (200)γ peak is the widest one. Therefore, this peak is the most “sensitive” for differences in wear conditions. In order to differentiate wear conditions, it is convenient to compare FWHM of (200)γ peaks of quenched samples after abrasive wear in investigated wear modes. Another way to characterize the differences in wear modes is to compare relative increment of FWHM for worn and as-quenched samples. According to Table 3, the relative increment in FWHM of (200)γ for the B1000 sample reaches 560%, which is 2.4 times greater than for the A1000 sample (237%);
- Because of phase transformation in the course of abrasive wear, there may be a case when γ peaks will be not suitable for calculating FWHM because of the little amount of retained austenite on the worn surface after wear. However, in any case of abrasive wear, prominent α peaks will be presented. Therefore, α peaks are more reliable to differentiate abrasive wear modes. According to the data in Table 2, the (200)α peak is the most “sensitive” one.

### 3.3. Microstructure of Subsurface Zones

**Figure 2:**
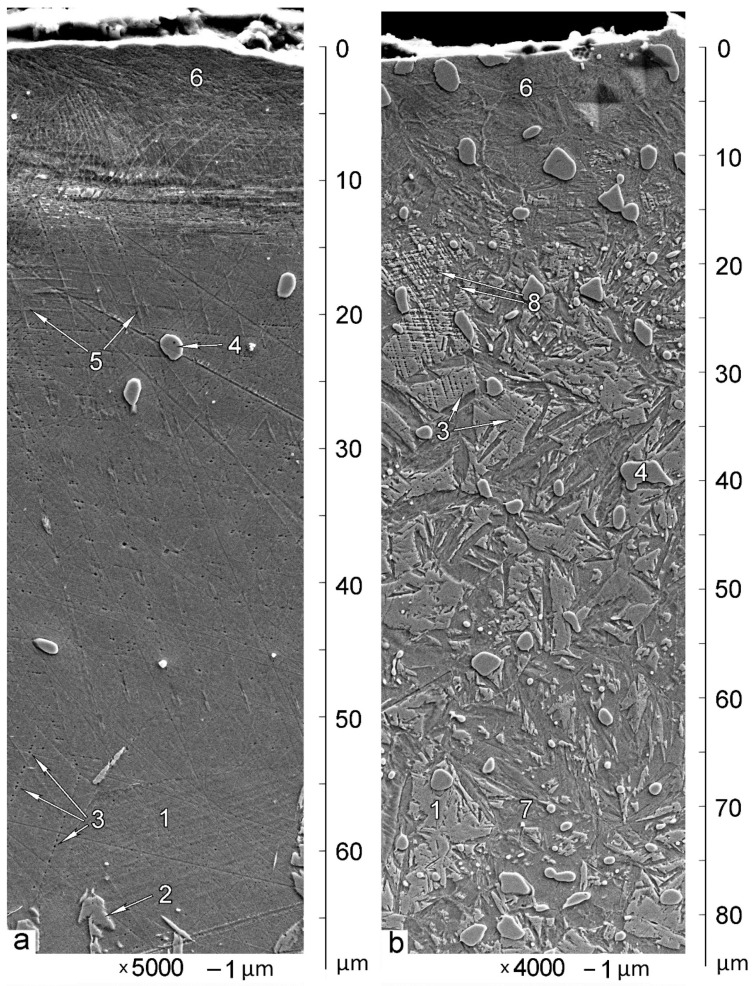
Microstructure of subsurface zones of X120Mn3Si2 steel after quenching and wear in B mode: (**a**) quenching from 1000 °C; (**b**) quenching from 900 °C; 1—austenite; 2—polishing-induced martensite; 3—etch pits; 4—carbides; 5—presumably transformation-induced martensite along slip planes; 6—surface zone formed by abrasive wear; 7—as-quenched martensite; 8—zone of ultimate plastic deformation of austenite. The prints in the upper right corner on image (**b**) were not used for microhardness calculation.

SEM micrographs of subsurface zones for B1000 and B900 samples are shown in Figure 2. Initial microstructures of X120Mn3Si2 steel in the as-quenched condition can be observed at the depth of 50–80 µm. Quenching from 1000 °C results in a predominantly austenitic state (i.e., 94% of γ-phase) (see Table 1, Figure 2a at depth 50–60 µm). Retained austenite (1) is the only structural constituent that is visible here. A minor quantity of undissolved carbides (4) are randomly distributed in the structure after quenching from 1000 °C, as can be seen in Figure 2a.

Since retained austenite is instable, it can undergo local martensitic transformation on the very surface of the microsection during grinding and polishing of the sample. This results in the formation of thin plates of surface martensite. Therefore, local areas of surface martensite (2) are observed in the SEM image.

Chains of etch pits (3) are observed in austenite (see Figure 2a). The less the depth, the more etch pits appear. They are located along crossing slip planes. This is the evidence of extensive plastic deformation due to abrasive wear. Some phase (5) appears along slip planes. It is presumably deformation-induced martensite. This martensite appears up to a depth of approximately 30 µm. The less the depth, the more martensite appears along slip planes. zone 6 of some specific grid-like microstructure appears at the very surface, up to about 10 µm depth.

Quenching of X120Mn3Si2 steel from 900 °C results in a microstructure that is significantly different. Undissolved carbides (4) are present in a decent quantity. Therefore, the carbon concentration in austenite before quenching is far less than 1.2%. It leads to an increasing of the M_s_ temperature. As a result, martensite (7) appears after quenching in a quantity of about 30%, while 60% of austenite (1) (see Table 1) remains in the structure.

This way, austenite volumes (1) are surrounded by as-quenched martensite (7). This martensite protects austenite from external stresses to a certain extent. Therefore, intensive plastic deformation of austenite may be observed far closer to the worn surface for X120Mn3Si2 steel quenched from 900 °C (see chains of etch pits (3) on Figure 2b at 30–35 µm depth). The closer to the surface, the closer are etch pits located to each other. At 20–25 µm depth, ultimate plastic deformation of austenite is reached (zone (8) on Figure 2b). This is where intensive mechanically induced martensite transformation begins. At the very surface, the zone (6) is formed, which is visually identical to that in Figure 1a. The only difference is the undissolved carbides.

**Figure 3:**
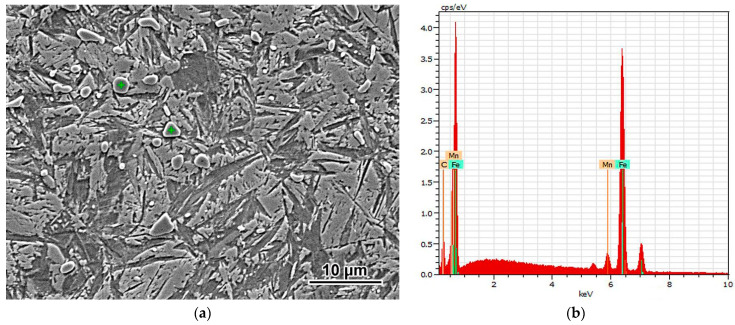
(**a**) The points of EDX analysis and (**b**) corresponding EDX spectrum (900 °C-quenched sample).

Since the XRD patterns did not present any carbide peak, an EDX study was employed to prove the existence of carbide in the steel quenched from 900 °C and 1000 °C. Point analysis performed on inclusions (as shown in Figure 3) revealed its average chemical composition as 8.46 ± 0.19 wt.% C, 5.92 ± 1.01 wt.% Mn, and 85.62 ± 1.06 wt.% Fe. According to these data, the inclusions can be identified as carbide (Fe, Mn)_3_C. The carbon content obtained was found to be higher than the stoichiometrical value of 6.67 wt.% for Fe_3_C. This discrepancy can be explained in view of the semi-qualitative character of EDX study, especially its high sensitivity to carbon contamination. Since the size of carbides was less than the EDX spatial resolution, the matrix affected the EDX result, leading to carbon content exaggeration.

**Figure 4:**
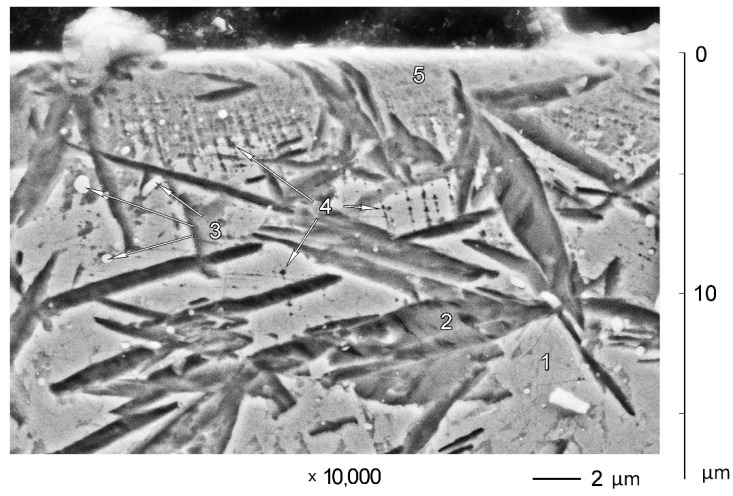
Microstructure of subsurface zones of X120Mn3Si2 steel after quenching from 900 °C and wear in A mode: 1—austenite; 2—martensite; 3—carbides; 4—etch pits; 5—surface zone formed by abrasive wear.

**Figure 5:**
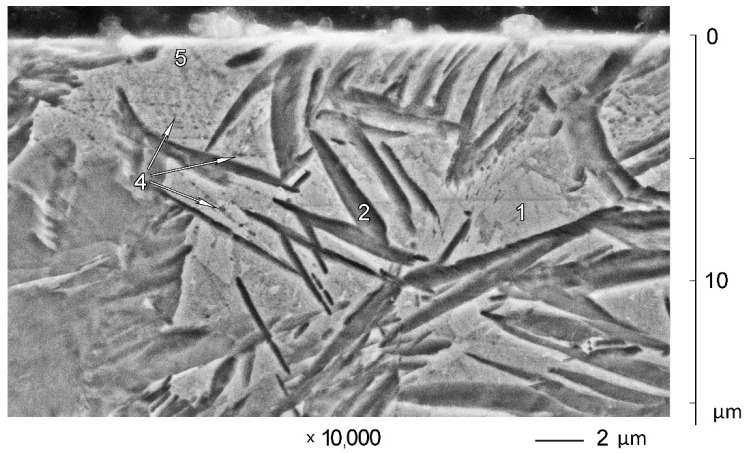
Microstructure of subsurface zones of X120Mn3Si2 steel after quenching from 1000 °C and wear in A mode: 1—austenite; 2—martensite; 4—etch pits; 5—surface zone formed by abrasive wear.

In contrast, the microstructure of subsurface zones of A900 (Figure 4) and A1000 (Figure 5) samples shows a much smaller depth of the plastically deformed layer. Etch pits (4) along slip planes are visible from about 10 μm distance to worn surface. The zone of deformed microstructure 5 is very thin (up to 3 μm), and it does not cover the entire worn surface.

Comparison of subsurface zone microstructures of A-mode and B-mode samples shows the general similarity and significant difference in details. Plastic deformation of austenite with subsequent transformation into mechanically induced martensite is the general similarity. The presence of martensite on the very surface is visible for both A-mode and B-mode samples (see Figure 2 and Figure 4). It is also confirmed by XRD investigation of worn surfaces (see Table 1). The biggest difference between A-mode and B-mode samples is the depths of plastically deformed layers and the depths of martensitic layers at the very surface. This is where the difference in abrasive wear conditions can be detected by the difference in microstructures of subsurface zones of worn samples.

### 3.4. Microhardness of Worn Surfaces

Measuring the microhardness of a worn material at a certain distance from the surface is a commonly used technique to demonstrate the gradient of microhardness of the friction surface in-depth [12,13,35,36,37,38,39]. The only drawback of this technique is that it is hard to measure the microhardness of the surface itself. 

**Figure 6:**
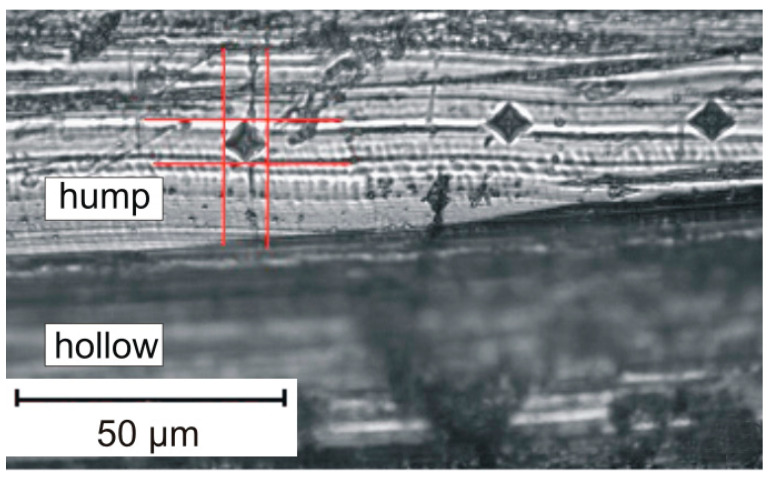
The technique for measuring the microhardness of a worn surface.

The same thought is expressed also in [40], with regards to how to overcome this obstacle. Not a cross-section, but the top surface is exposed to microhardness indentations. Despite the high surface roughness, small surface areas exist with very low roughness. Therefore, it is possible to make one or even several indents to measure microhardness (Figure 6). One minor problem is distinguishing humps from hollows and making indentations only on humps. This distinction can be made by a smooth approaching of the microscope lens to the surface. The humps are focused first, while hollows remain blurred (see Figure 6).

**Figure 7:**
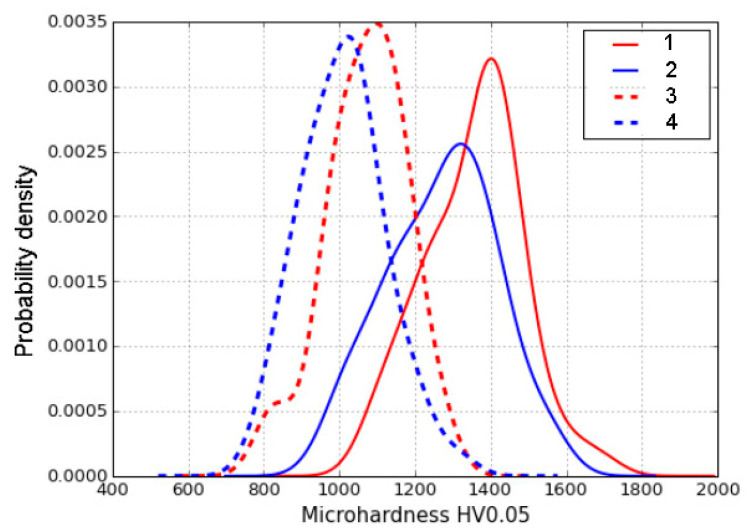
Distribution of worn surface microhardness for samples worn in A and B modes: 1—B900; 2—B1000; 3—A900; 4—A1000.

The microhardness of a worn surface is not a constant because of the fatigue nature of wear. Therefore, a relatively broad range of microhardness values is expected for each worn sample. From 50 to 60 indents have been made on every sample to get the microhardness distribution. The result is shown in Figure 7. 

According to the results obtained, it is possible to make some general conclusions:

- The microhardness values of the worn surface of B-mode samples are noticeably greater than those of A-mode samples;
- The microhardness of the worn surface of all samples is higher than that of many industrial-wear-resistant hardfacings [41], and at the same level or higher than that of chromium carbide plasma fabricated coatings [42,43].

Values of microhardness at the level of 1000–1100 HV0.05 (like those obtained on A-mode samples) are generally accepted for mechanically induced martensite which appears from instable retained austenite in the course of abrasive wear [44]. Values in the range of 1300–1400 HV0.05 (B-mode samples) are higher than expected. The difference in surface microhardness between worn surfaces of A-mode samples and B-mode samples may be explained by different pressure in different wear modes. Pressure in the B wear mode is higher than that in the A wear mode. This is the reason for the difference in the phase composition of the worn surface (see Table 3). The less austenite in the worn surface (Table 3), the higher the hardness (Figure 7).

Assuming FWHM of (200)α peaks as indicators of difference in abrasive wear conditions (see Section 3.2), it is necessary to check the correlation of this parameter with the microhardness of the worn surface. According to Figure 7, the values of microhardness in maximums are approximately 1000 HV0.05, 1100 HV0.05, 1300 HV0.05, and 1400 HV0.05 for A1000, A900, B1000, and B900, respectively. Corresponding values of (200)α peaks are (in degrees): 2.27, 1.64, 3.20, and 3.28 (see Table 2). The linear correlation coefficient for these values is equal to 0.83. This value may be interpreted as an existing linear correlation between the FWHM of the (200)α peak and the microhardness of the worn surface. For the (211)α peak, the similar correlation coefficient is equal to 0.88. In order to get better correlation coefficients, it is necessary to perform a more comprehensive investigation where a wider set of wear conditions would be considered.

### 3.5. Worn Surface Examination

**Figure 8:**
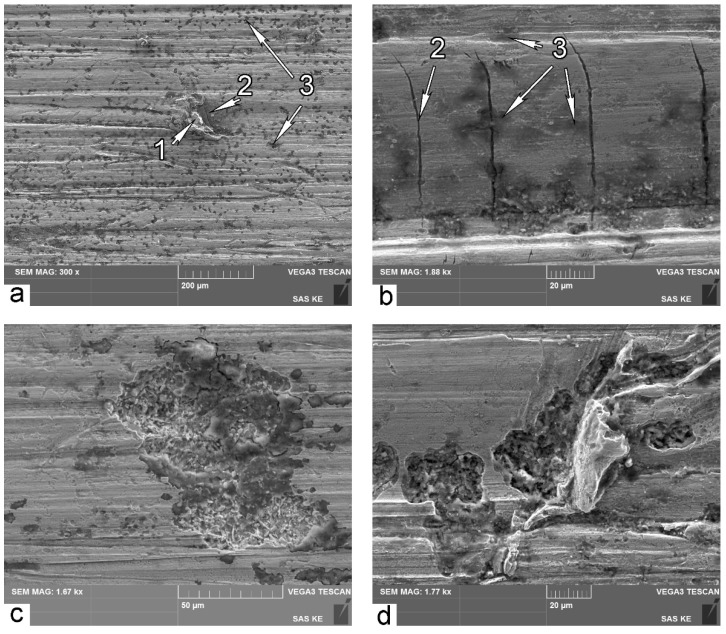
SEM micrographs of surface after wear in B mode: (**a**,**b**,**d**) B1000 sample; (**c**) B900 sample; 1—embedded abrasive particle; 2—transverse microcracks; 3—spots of fatigue damage.

Figure 8 illustrates typical SEM micrographs of the worn surface for B-mode samples. Small magnifications reveal general features of the worn surface after three-body abrasive wear (Figure 8a). Embedded abrasive particles 1 are extensively distributed over the worn surface. Transverse microcracks 2 are also a distinctive feature for B-mode samples. These cracks indicate brittle failure; it means that the material has reached its ultimate possible hardening.

If the material reaches the utmost possible hardening, then no further plastic deformation is possible. In this case, high-cycle fatigue wear occurs. It was supposed that spots 3 (Figure 8a,b) indicate the sites that are in a pre-failure state due to high-cycle fatigue. It should be noted that these spots are darker than other places on the surface. Because of that, the surface area of darker points on SEM micrographs of the worn surface can be used as indicators for portions of high-cycle failure mode in the overall spectrum of wear mechanisms.

Figure 8c shows a typical site of high-cycle fatigue failure after detaching the chip of material. Such a site is similar to failure pitting points on bearing tracks damaged by contact fatigue [45]. Figure 8d illustrates the event of detaching the chip of material from the worn surface. Figure 8c,d prominently illustrate sites of fatigue damage. This can serve as the proof of the assumption that darker spots on Figure 8a,b indicate the sites that are in a pre-failure state due to high-cycle fatigue.

High-cycle fatigue wear takes place only if the contact stress is lower than the yield stress of the material. It is obvious that the yield stress of the material on the very friction surface is significantly higher than that in the bulk. This is due to significant initial plastic deformation of the subsurface in the course of abrasive wear, extensive γ-α transformation, and significant rise of microhardness.

**Figure 9:**
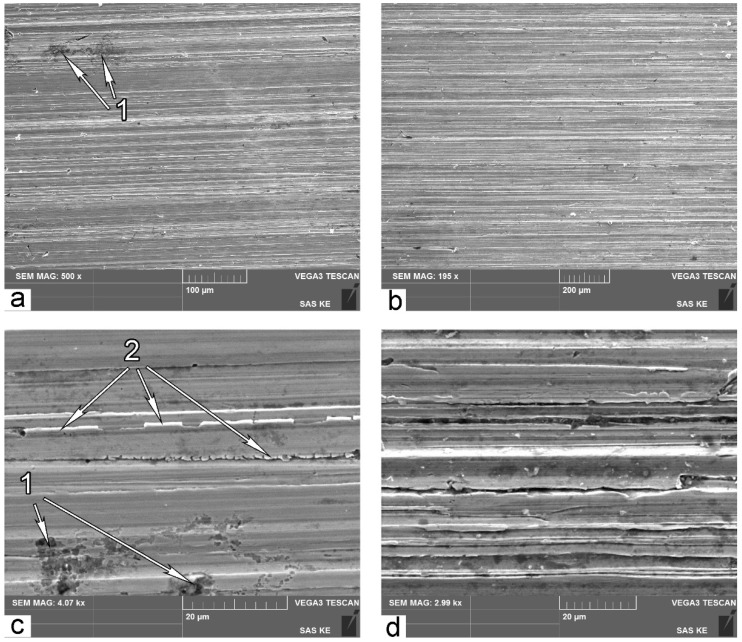
SEM micrographs of surface after wear in A mode: (**a**,**c**) A900 sample; (**b**,**d**) A1000 sample: 1—spots of fatigue damage; 2—cracking of ridges.

In contrast, SEM images of worn surface after wear in A mode (Figure 9a,b) show far fewer sites of high-cycle fatigue 1. This is because low-cycle fatigue and/or microcutting are predominant mechanisms of surface failure in A wear mode. Images in higher resolution reveal typical features of failure in these modes: cracking of ridges 2 (low-cycle fatigue) and formation of deep grooves (microcutting) as shown in Figure 9c,d.

According to worn surfaces examination, it is evident that fatigue rupture is the leading wear mechanism for the B wear mode, and microcutting appears to be much more probable for the A wear mode. A combination of different leading wear modes with different loads may be considered as an explanation of the different behaviors of the same material in different abrasive wear conditions.

The results of the present study can be used for different industrial applications for increasing the life span of the machine parts exploited under severe abrasion. Specifically, it shows how important it is to tailor the austenite wear response (through controlling the deformation martensite transformation kinetic via heat treatment regime) depending on particular wear conditions (abrasive hardness, load, etc.). Exploiting the metastability of austenite in cheap low-alloyed steel allows involving the internal reserve of material to withstand the abrasion due to the TRIP effect, thus leading to significant cost-saving in different industrial processes (mineral comminuting, pulp transportation, coal, steel scrap briquetting, etc.).

## 4. Conclusions

- Three distinguished areas of structure patterns may be observed at the cross-sections of under-surface regions of the B samples after abrasive wear. The area of the very beginning of plastic deformation is visible approximately 60–30 μm below the surface. This area is characterized by multiple slip, which is visible due to etch pits decorating slip planes. The area of plastic deformation gradually transforms to a uniform area of transformed material under 10–15 μm depth. The structure of samples worn in A mode demonstrates the very beginning of plastic deformation not deeper than 10 μm below worn surface;
- The microhardness of the worn surface of samples that are worn in B mode is noticeably higher than for samples that are worn in A wear mode. The highest microhardness measured exceeds 1400 HV0.05 for B900 samples;
- X-ray investigations revealed differences between A and B worn surfaces which correlate with difference in wear modes, material structure, and microhardness. Values of FWHM for all corresponding standing peaks for both α and γ phases alone are bigger for B samples than for A samples. It is also shown that the (200) peaks for α and γ got the most widening in every given wear mode. Considering α and γ peaks as indicators of abrasive wear “severity”, it is shown that α peaks are more suitable because they are always present on diffractograms of the worn surface of tested steel because of more or less prominent γ-α transformation. At that γ, peaks are not always sufficiently intensive in comparison with background noise, and therefore it is not possible to calculate the FWHM of those peaks with acceptable accuracy. This may happen if wear is severe enough to cause full γ-α transformation (for example, in B wear mode);
- Sites on the friction surface that are under the high-friction fatigue mode of rupture appear darker than the rest of surface when observed in SEM. This fact can be used as an additional indicator of the wear failure mode of the friction surface, and in particular, wear conditions;
- Quenching from 900 °C for X120Mn3Si2 steel is a more favorable treatment for practical use because the work-hardening of the friction surface achieves its maximum in this case;
- Samples quenched from 1000 °C may be used as detectors of wear mode. Even in conditions of abrasive wear, which is the most aggressive among all types of wear, this structure is sensitive enough to reveal differences in wear conditions via the different phase compositions, microhardness values, and microstructures of the friction surface. This sensitivity is achieved due to austenite that is a bit more stable in comparison with that after quenching from 900 °C. Increased stability leads to increased “sensitivity” of the structure to the severity of loading conditions in friction contact. The less austenite remains in the surface after wear, the more severe wear conditions are. This may be useful for express estimation of wear conditions in practice. Further research is needed to determine the scope of practical implementation for the retained austenite as a sensitive structure to differentiate wear conditions.

## Figures and Tables

**Table 1:** Results of abrasive wear tests.

Sample Designation	Wear Path, m	Mass Loss, mg	Wear Rate, mg/m
A900	96	47.6	0.50
A1000	96	56.9	0.59
B900	8	215.4	26.9
B1000	8	198.7	24.8

**Table 2:** FWHM, 2Θ deg., for investigated samples.

Sample Designation	α Peaks		γ Peaks		
	(200)	(211)	(200)	(220)	(311)
Quenching from 800 °C (reference)	1.34	1.23	-	-	-
Quenching from 1000 °C (reference)	-	-	0.30	0.37	0.54
B1000	3.20	2.62	1.98	1.03	1.45
A1000	2.27	1.77	1.01	0.98	1.05
B900	3.28	2.41	-	-	-
A900	1.64	1.75	-	-	-

**Table 3:** Changing FWHM and content of γ-phase in worn samples relative to as-quenched state.

Sample Designation	∆FWHM				Volume Fraction % of γ-Phase		∆% of γ-Phase
	(200) α		(200) γ		as-Quen-ched	Worn Surface	
	Absolute, 2Θ, °	Relative, %	Absolute, 2Θ, °	Relative, %			
B1000	1.86	139	1.68	560	93.9 [26]	10.2	83.7
A1000	0.93	69	0.71	237		19.7	74.2
B900	1.94	145	-	-	60.1 [26]	4.3	55.8
A900	0.30	22	-	-		12.5	47.6

## Data Availability

The data presented in this study are available on request from the corresponding author.
